# Supplementary material for: Diverse and asymmetric patterns of single-neuron projectome in regulating interhemispheric connectivity
Source: Nat Commun. 2024 Apr 22;15:3403. doi: 10.1038/s41467-024-47762-y (PMC11035633; doi:10.1038/s41467-024-47762-y)
Supplement: Supplementary file 1 — Supplementary Information [file 41467_2024_47762_MOESM1_ESM.pdf]

Diverse and Asymmetric Patterns of Single-Neuron Projectome in Regulating Interhemispheric Connectivity

Yao Fei[1,2,#], Qihang Wu[2,3,#], Shijie Zhao[1,4,*], Kun Song[2,3], Junwei Han[1,4,*], Cirong Liu[2,3,5,*]

[1]School of Automation, Northwestern Polytechnical University, Xi'an, China;

[2]CAS Center for Excellence in Brain Science and Intelligence Technology, Institute of Neuroscience, Chinese Academy of Sciences, Shanghai 200031, China.

[3]University of Chinese Academy of Sciences, Beijing 100049, China

[4]Research & Development Institute of Northwestern Polytechnical University in Shenzhen, Shenzhen, China.

[5]Key Laboratory of Genetic Evolution & Animal Models, Chinese Academy of Sciences, China

[#] These authors contributed equally

*Co-corresponding authors

Dr. Cirong Liu, E-mail: crliu@ion.ac.cn; Dr. Junwei Han, E-mail: jhan@nwpu.edu.cn; Dr. Shijie Zhao, E-mail: shijiezhao666@gmail.com

Supplementary Figures

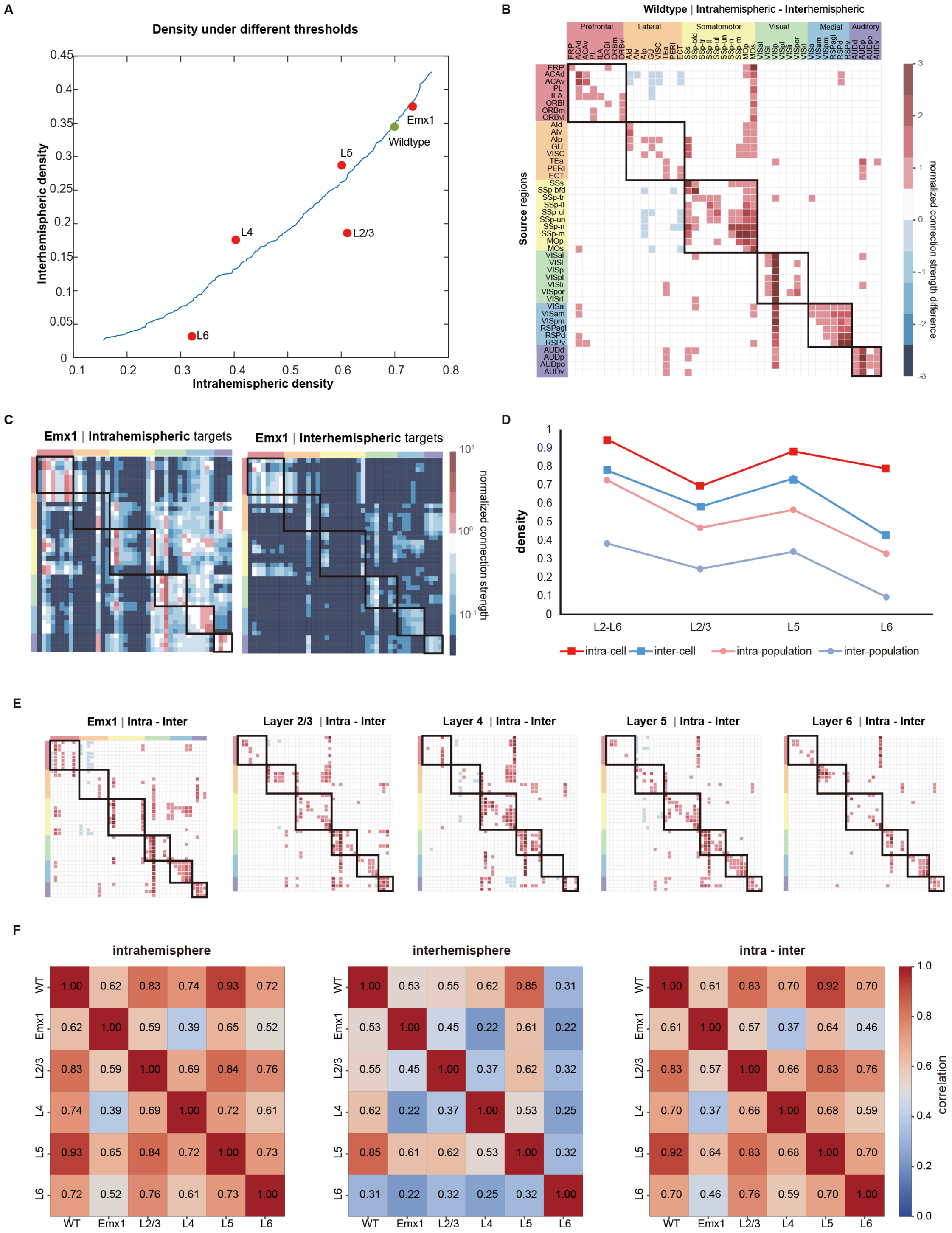

Supplementary Figure 1. Supplementary results on structural connectivity data. (A) The connectivity density of intrahemispheric and interhemispheric connections under varying thresholds is shown. The blue line represents the density of wild-type mice from high threshold (low density) to low threshold. The circles illustrate the density of different mouse strains under the default threshold. **(B)** Contrast matrix derived from wild-type mice. (**C**) Connectivity matrices derived from Emx1 mice. **(D)** Connection density across different layers. "intra-cell" and "inter-cell" represent intrahemispheric and interhemispheric connections from ION single-neuron data, while "intra-population" and "inter-population" represent connections from Allen population data, respectively. **(E)** Contrast matrices generated from Emx1 mice and layer-specific Cre mice data. **(F)** The correlation among different layers for intrahemispheric connections, interhemispheric connections, and contrast matrices. Source data are provided as a Source Data file.

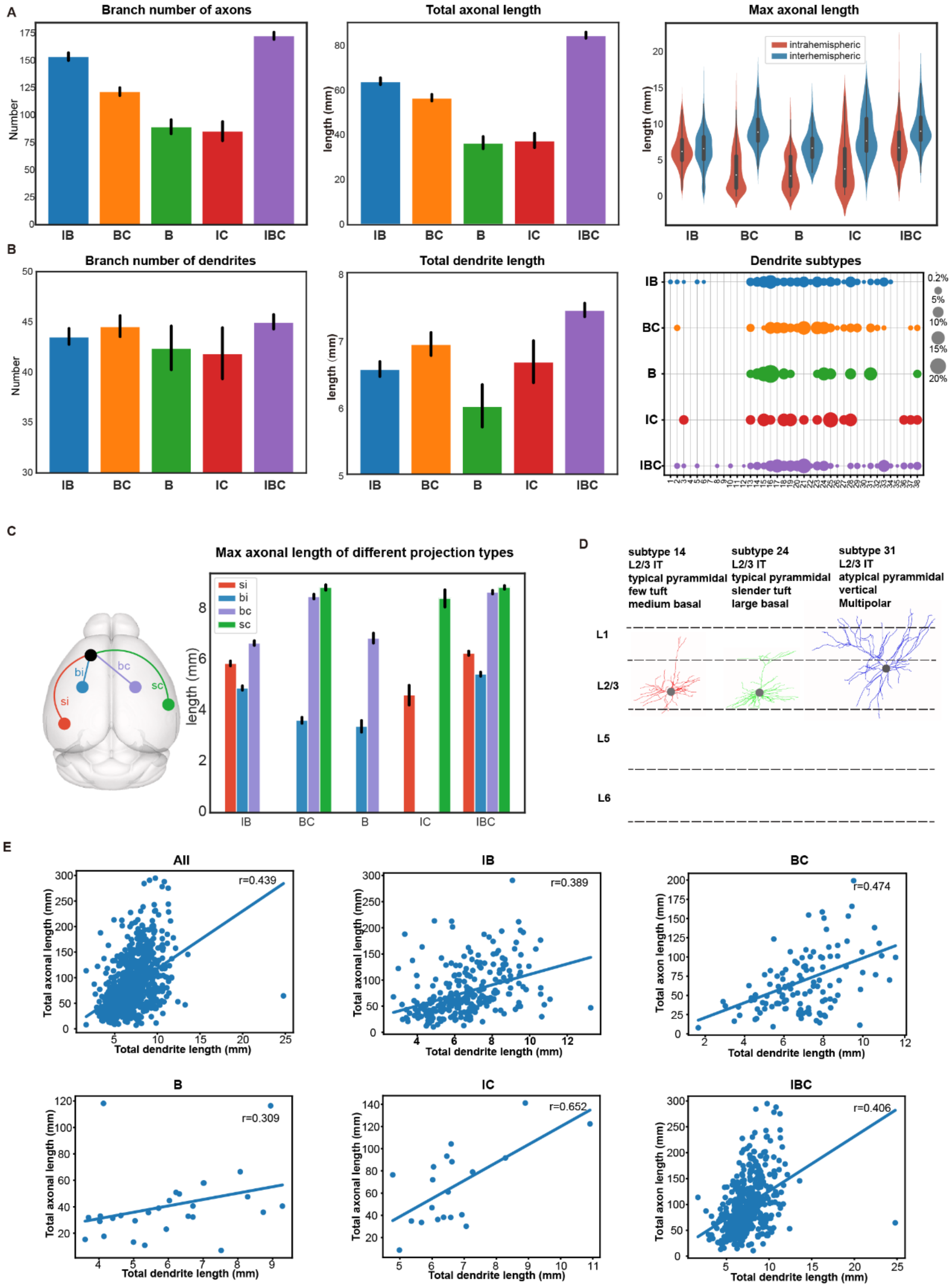
A
Branch number of axons
Number
IB
BC
B
IC
IBC
Total axonal length
length (mm)
Max axonal length
intrahemispheric
interhemispheric
B
Branch number of dendrites
Total dendrite length
Dendrite subtypes
0.2%
5%
10%
15%
20%
C
Max axonal length of different projection types
si
bi
bc
sc
D
subtype 14
L2/3 IT
typical pyrammidal
few tuft
medium basal
subtype 24
L2/3 IT
typical pyrammidal
slender tuft
large basal
subtype 31
L2/3 IT
atypical pyrammidal
vertical
Multipolar
L1
L2/3
L5
L6
E
All
r=0.439
Total axonal length (mm)
Total dendrite length (mm)
IB
r=0.389
BC
r=0.474
Total axon length (mm)
B
r=0.309
IC
r=0.652
IBC
r=0.406

Supplementary Figure 2. Supplementary morphological results on the single-neuron projectome. (A) Axonal morphologies of different neuronal types, including the number of branches (left), the total axonal length (middle), and the maximum axonal length (right) of neurons. Bar plots present data as mean ± SEM which are provided in Source data. For maximum axonal length, intrahemispheric and interhemispheric axons are estimated separately. Data are presented by the violin plot and the median value, the 25th percentile, the 75th percentile, the maximum value, and the minimum value of the violin plots are provided in **Supplementary Data 8**. **(B)** Dendritic morphologies of different neuronal types, including the number of branches (left), the total dendritic length (middle), and the dendritic subtypes (right) of neurons. The dendritic subtypes were defined by a previous study[38], which classified dendrites into 38 subtypes based on their morphologies. **(C)** Maximum axonal length for different projection types, including axons projecting to a specific intrahemispheric target (si), axons projecting to an intrahemispheric target with a common heterotopic target (bi), axons projecting to a specific heterotopic target (sc), and axons projecting to a heterotopic target with a common intrahemispheric target (bc). Data are presented as mean ± SEM. **(D)** Three subtypes of dendrites are present in neurons with bilateral projections (IB, BC, B, and IBC) but absent in IC neurons. The corresponding neuron IDs of three dendrite types illustration are provided in **Supplementary Data 4**. **(E)** Correlation between total axonal length and total dendrite length in different neuron types, with IBC neurons having the longest axonal and dendritic projections. The statistical analysis results of **(A-C)** are provided in **Supplementary Data 5**. Source data of (A-C, E) are provided as a Source Data file.

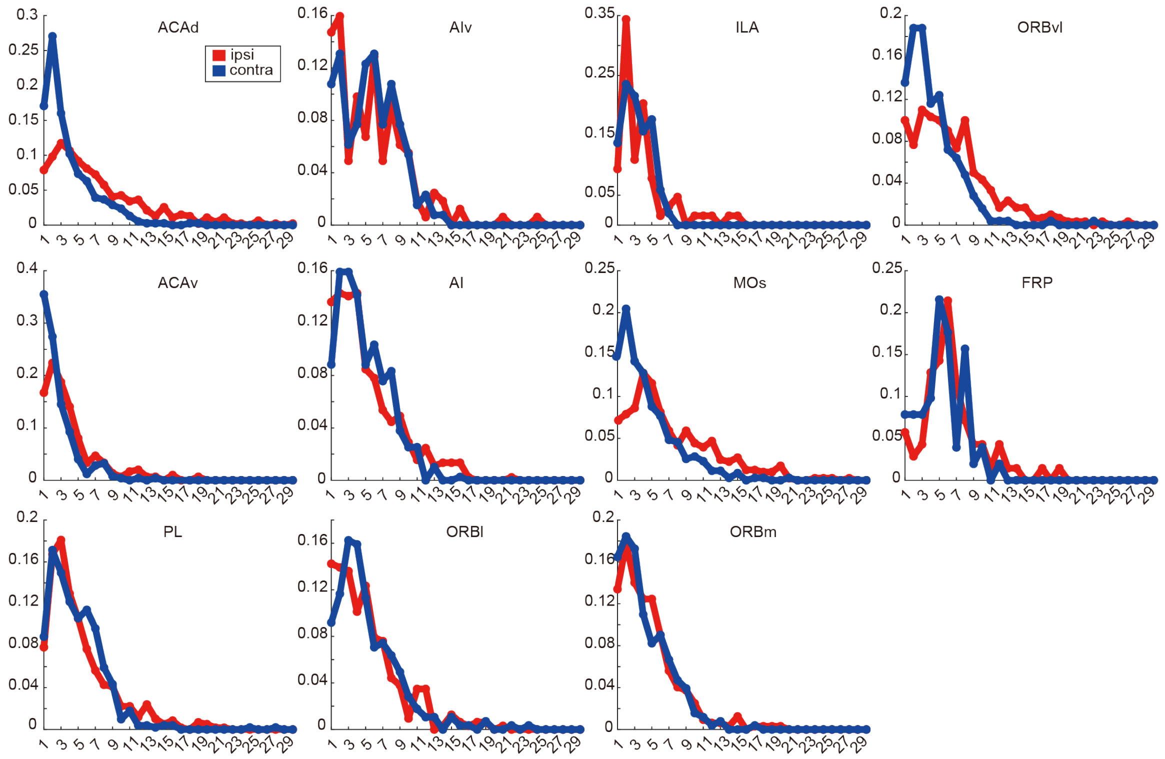

Supplementary Figure 3. Heterogeneity of projections to multiple heterotopic downstream from 11 PFC upstream regions. The red lines are the heterogeneity of ipsilateral projections, and the blue lines represent contralateral projections. Source data are provided as a Source Data file.

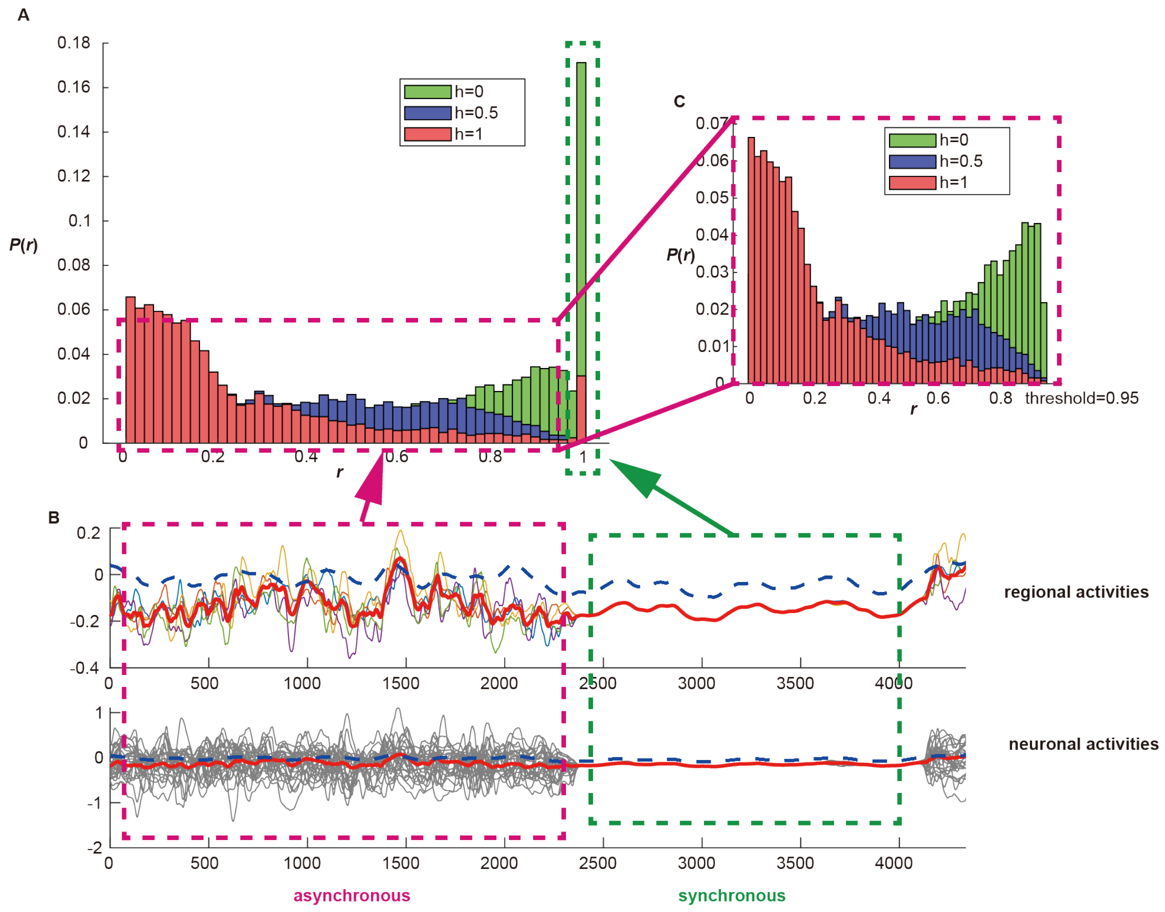

Supplementary Figure 4. Defining asynchronous and synchronous states. (A) The distribution of inter-regional correlation at different time points exhibits two parts: the large correlation values that are close to one (inside the green dashed box) and the other smaller correlation values (inside the pink dashed box). We set a threshold of correlation values to separate them. The distribution under three different levels of heterogeneity were compared (green~h=0, blue~h=0.5, red~h=1). (B) The two groups of correlation values in (A) correspond to two periods of the network dynamics: asynchronous periods and synchronous periods. In the upper row, the red line is the mean activity of the whole network, and the other colorful lines represent the activities of different regions. In the bottom row, the gray lines are activities of randomly selected neurons and the blue dashed line is the noise external input. (C) The

detailed distribution of correlation values during the asynchronous periods, corresponding to the pink dashed box in (A). Source data of (A-C) are provided as a Source Data file.

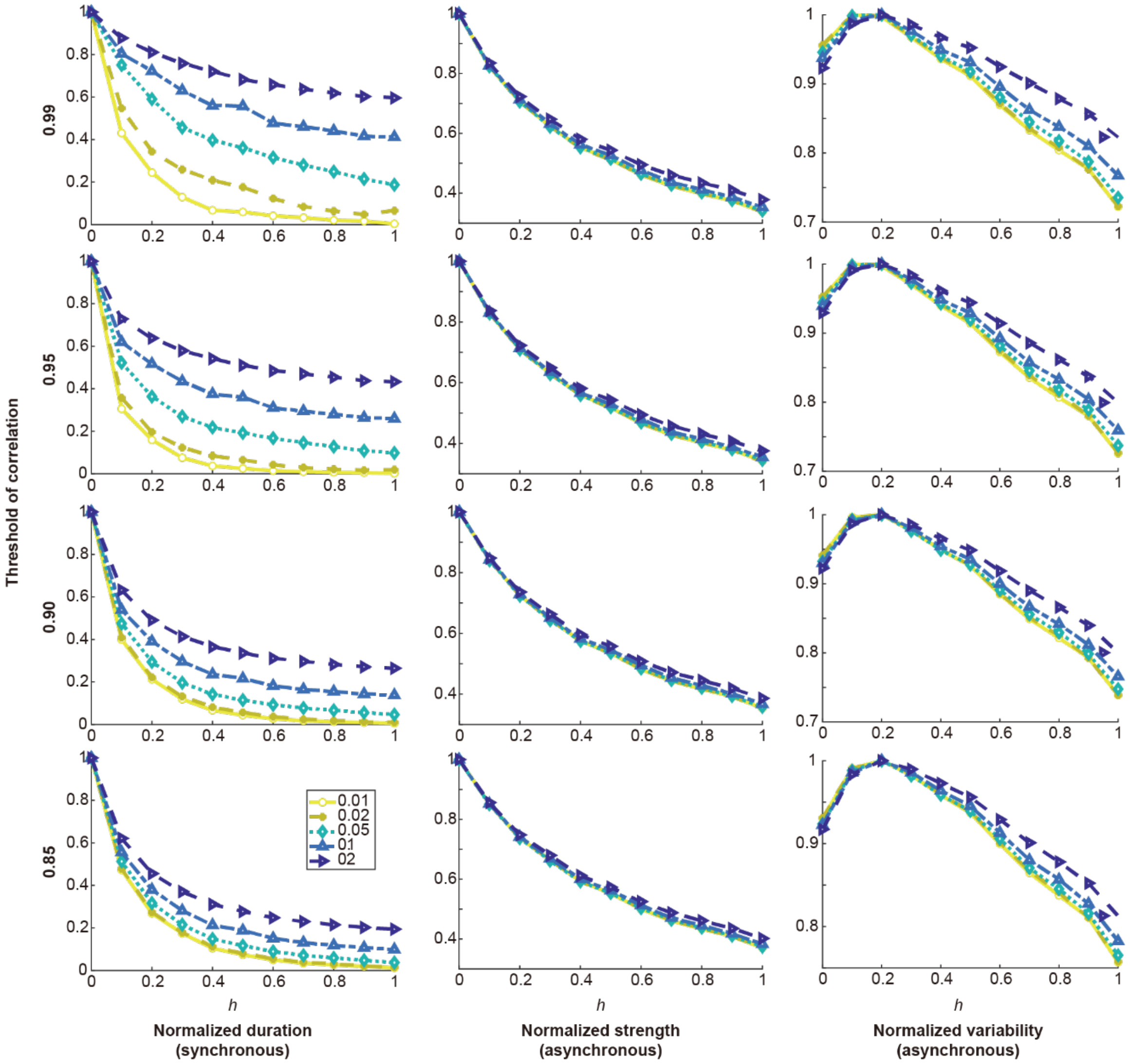

Supplementary Figure 5. Duration of synchronous periods, and correlation strength and variability of asynchronous periods under different thresholds. Each row contains results under a certain threshold (0.85, 0.9, 0.95, 0.99). The x-axis of each sub-figure is the parameter h. The y-axis of the sub-figures in the first column is the normalized duration of synchronous periods and is the normalized strength and normalized variability for the second and third columns. Different levels of thresholds for defining states do not affect how heterogeneity changes the stability of the dynamics, as well as the strength and variability of correlation.

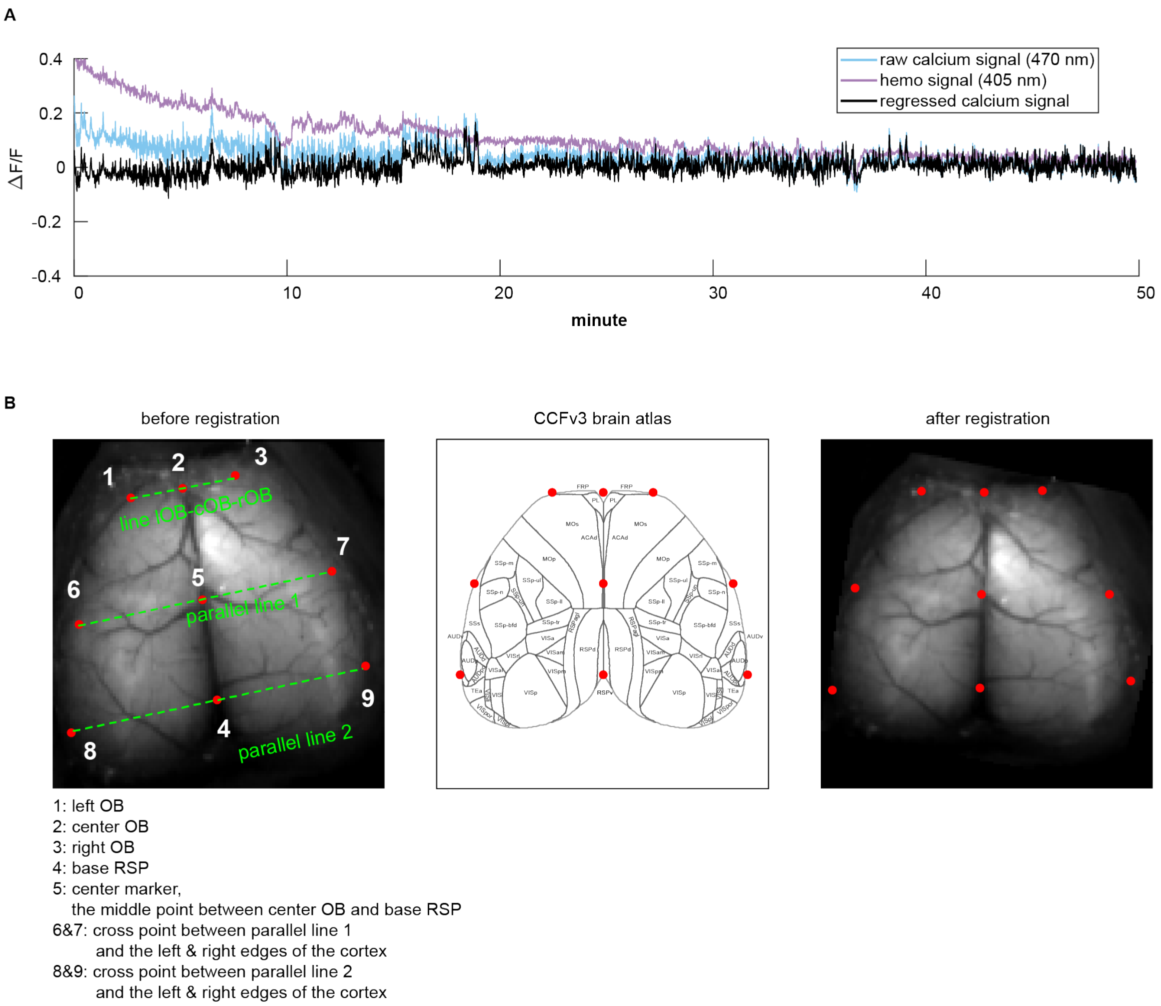

Supplementary Figure 6. Implementation of hemodynamics correction and CCFv3 brain atlas registration. (A) Hemodynamics correction. This panel illustrates the raw calcium imaging signal under 470 nm excitation light, the hemodynamics signal under 405 nm excitation light, and the regressed calcium signal. The descending trend characteristic of the hemodynamics is effectively corrected in the regressed calcium signal. **(B)** CCFv3 brain atlas registration. The left panel displays a frame from the widefield imaging before registration, the middle panel presents the CCFv3 brain atlas, and the right panel is the result of registering the raw frame to the CCFv3 atlas using affine transformation. Nine markers (depicted as red dots) are manually annotated on each frame, serving as the basis for calculating

the transformation matrix. Markers 1,2,3 are the left olfactory bulb (OB), center OB, and right OB. Marker 4 is the retrosplenial cortex (RSP). These four markers have clear anatomical locations. Marker 5 is the middle point of cOB and RSP. Markers 6 and 7 are the cross points between the line passing marker 5 parallel to line lOB-cOB-rOB (parallel line 1) and the left and right edges of the cortex. Similarly, markers 8 and 9 are cross points between parallel line 2 and the cortex edges. Source data of (A-B) are provided as a Source Data file.

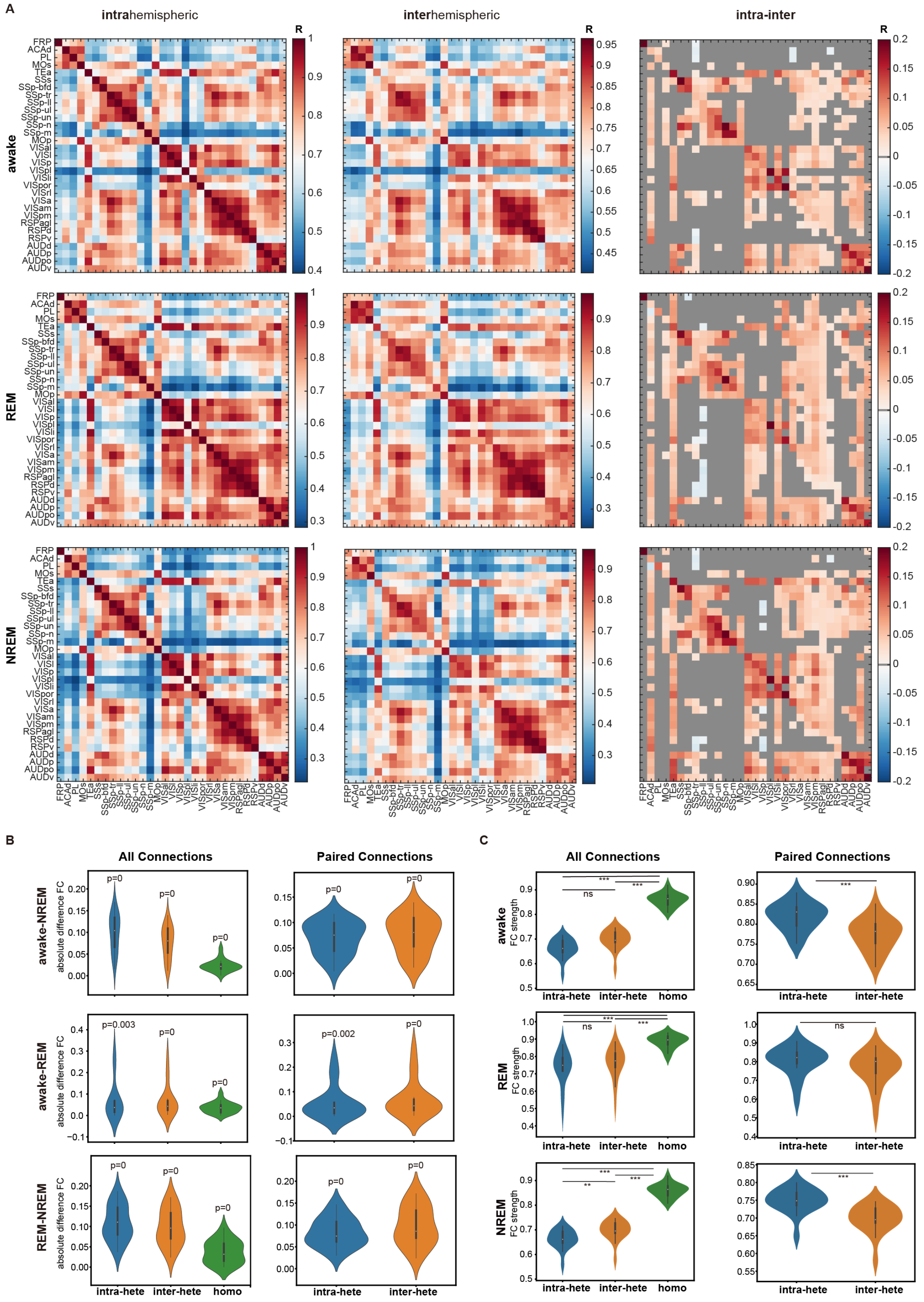
A
intrahemispheric
interhemispheric
intra-inter
R
awake
REM
NREM
FRP
ACAd
PL
MOs
TEa
SSs
SSp-bfd
SSp-tr
SSp-ll
SSp-ul
SSp-un
SSp-n
SSp-m
MOp
VISal
VISl
VISp
VISpl
VISli
VISpor
VISrl
VISa
VISam
VISpm
RSPagl
RSPd
RSPv
AUDd
AUDp
AUDpo
AUDv
B
All Connections
Paired Connections
awake-NREM
awake-REM
REM-NREM
absolute difference FC
p=0
p=0.003
p=0.002
intra-hete
inter-hete
homo
C
awake
REM
NREM
FC strength
ns
***
**

Supplementary Figure 7. **Supplementary results on wide-field data without global signal subtraction. (A)** The three matrices depict the functional connectivity of intrahemispheric connections, interhemispheric connections, and the difference between intrahemispheric and interhemispheric connections for awake, REM, and NREM sleep states from data without removing global signals. The gray areas of the third column represent connections where the FC strength differential is not statistically significant (n=19, a two-sided t-test, $p > 0.05$). The corresponding t-statistic and p-value for all connections are provided in **Supplementary Data 7**. (**B**) The distribution of absolute values of FC strength differences across the three types of connections-intrahemispheric-heterotopic, interhemispheric-heterotopic, and homotopic connections between any two states (N=19, a two-sided t-test). The p-value is less than 0.05, indicating that the difference is significant. The corresponding t-statistic and p-value for three types of connections are provided in **Supplementary Data 7**. The left column presents the results of functional connectivity from all anatomically connected regions, which have more intrahemispheric connections than heterotopic connections. The right compares interhemispheric-heterotopic connections with their corresponding intrahemispheric-heterotopic counterparts. **(C)** Functional connectivity strength of the three types of connections in different brain states (N=19, a two-sided Tukey-HSD test for multiple comparisons). ‘ns’, not significant; ‘*’, p<0.05; ‘**’, p<0.01, ‘***’, p<0.001. The median value, the 25th percentile, the 75th percentile, the maximum value, and the minimum value of all violin plots and adjusted p values for multiple comparisons are provided in **Supplementary Data 8**. Source data of (A-C) are provided as a Source Data file.

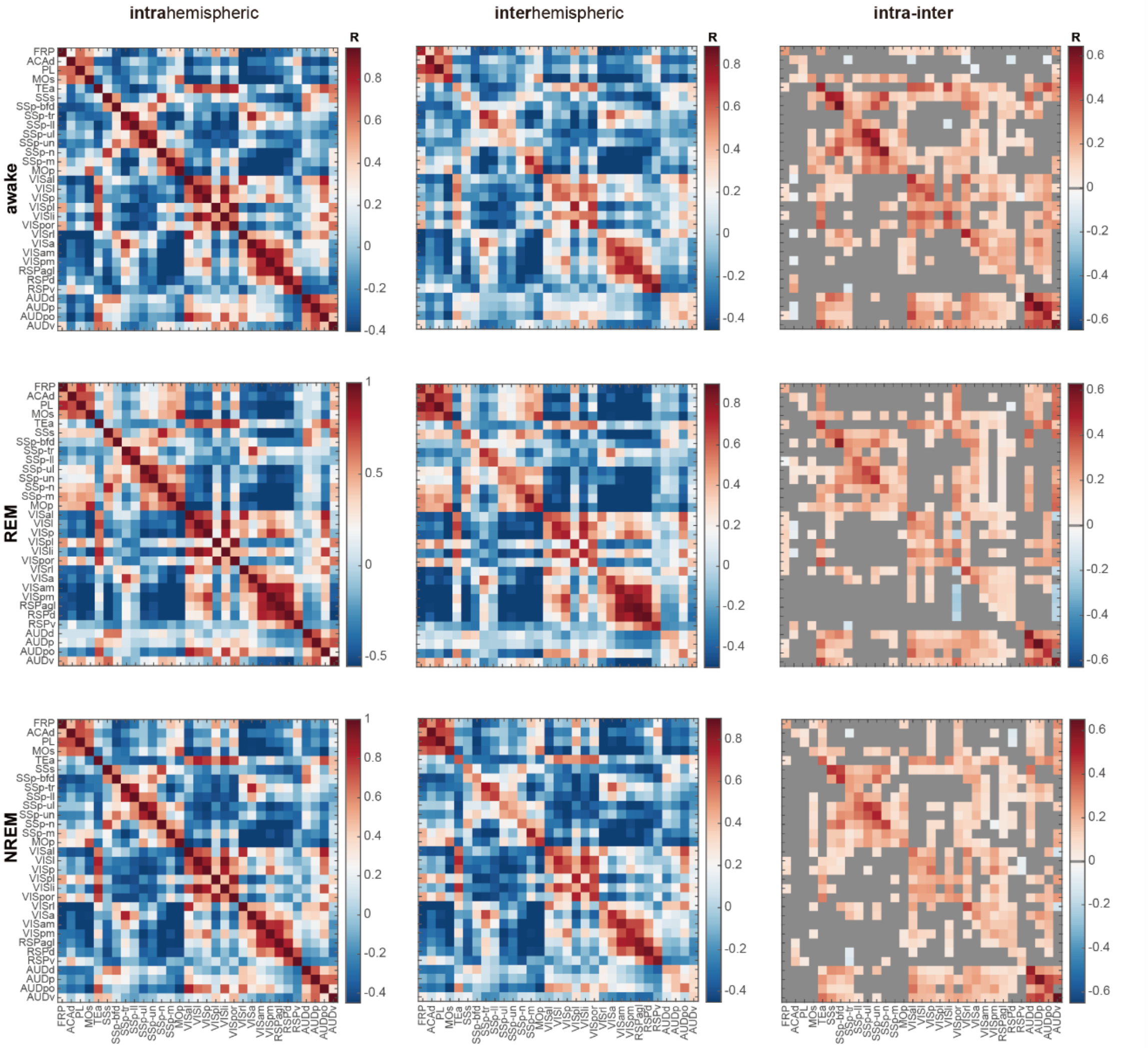

Supplementary Figure 8. FC matrices from wide-field data with global signal subtraction. The global signal subtraction was achieved by subtracting the mean time series of all pixels from the time series of each pixel. The three matrices depict the functional connectivity of intrahemispheric connections, interhemispheric connections, and the difference between intrahemispheric and interhemispheric connections for awake, REM, and NREM sleep states. The gray areas of the third column represent connections where the FC strength differential is not statistically significant (N=19, a two-sided t-test,

$p > 0.05$). The corresponding t-statistic and p-value for all connections are provided in **Supplementary Data 7**. Source data are provided as a Source Data file.

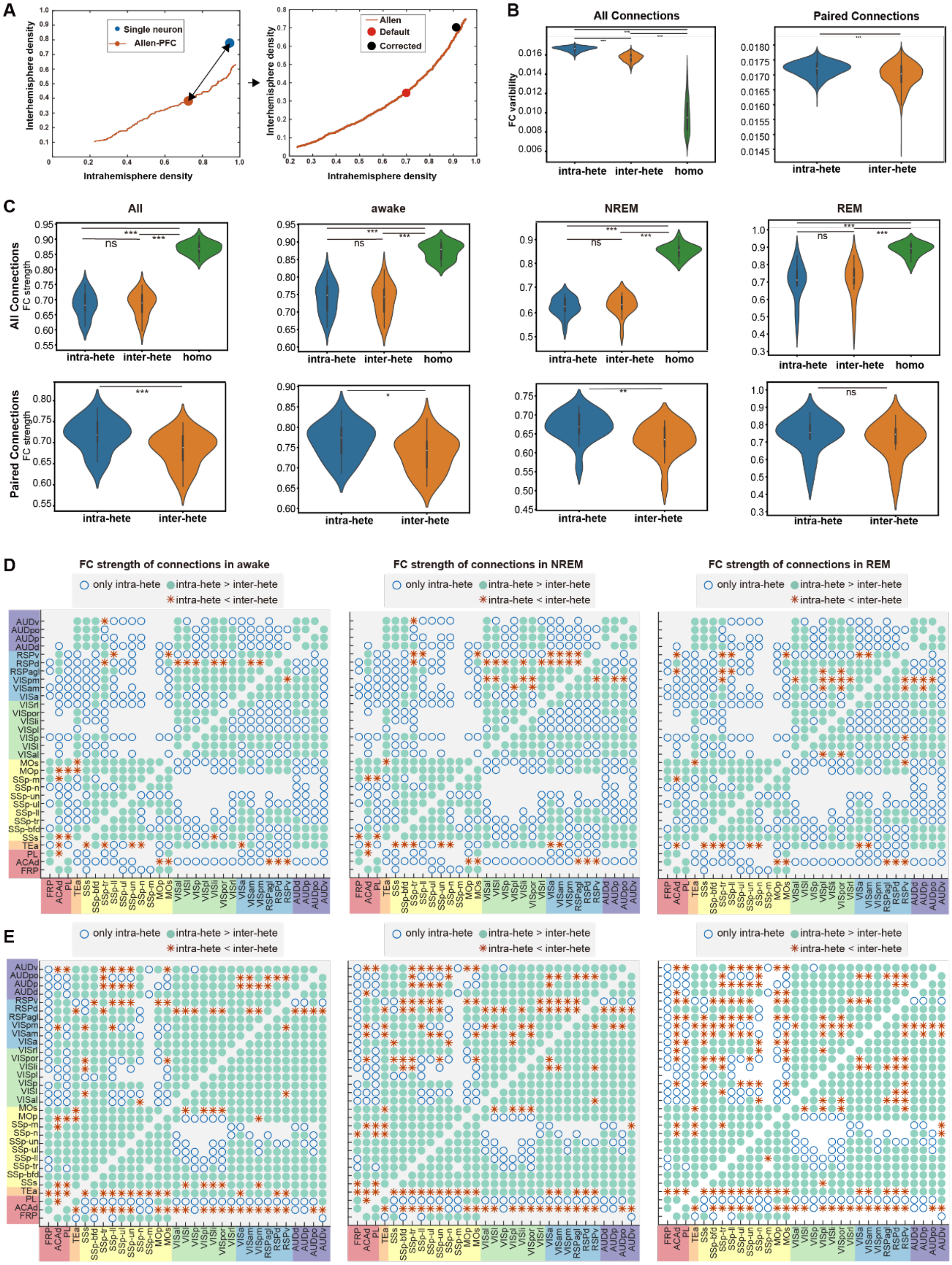
A
Single neuron
Allen-PFC
Interhemisphere density
Intrahemisphere density
Allen
Default
Corrected
B
All Connections
Paired Connections
FC varibility
intra-hete
inter-hete
homo
C
All
awake
NREM
REM
All Connections
FC strength
Paired Connections
ns
D
FC strength of connections in awake
FC strength of connections in NREM
FC strength of connections in REM
only intra-hete
intra-hete > inter-hete
intra-hete < inter-hete
E

1

Supplementary Figure 9. Supplementary results on the strength and stability of interhemispheric functional connectivity. (A) Threshold adjustment for the Allen population data. The left panel displays the density of the PFC projectome of Allen population data (orange) and ION single-neuron data (blue). For the Allen population data, the line represents the density under varying thresholds, and the dot indicates the density under the default threshold. In comparison with ION single-neuron tracing from the prefrontal cortex, Allen population data underestimate network density by 22.08% for intrahemispheric connections and 39.53% for interhemispheric connections. The right panel illustrates the density of all brain regions under the default and the adjusted threshold, based on the calculated ratio of underestimation. **(B)** Functional connectivity variability of the three types of connections under the adjusted threshold (N=1000, a two-sided Tukey-HSD test for multiple comparisons). **(C)** Functional connectivity strength of different brain states under the adjusted threshold (N=19, a two-sided Tukey-HSD test for multiple comparisons). 'ns', not significant; '*', p<0.05; '**', p<0.01, '***', p<0.001. **(D-E)** Functional connectivity strength of each connection in different brain states under the default threshold (D) and the adjusted threshold (E), respectively. The median value, the 25th percentile, the 75th percentile, the maximum value, and the minimum value of all violin plots and adjusted p values for multiple comparisons are provided in **Supplementary Data 8**. **(A-D)** Source data are provided as a Source Data file.
