# Supplementary material for: Diverse and asymmetric patterns of single-neuron projectome in regulating interhemispheric connectivity
Source: Nat Commun. 2024 Apr 22;15:3403. doi: 10.1038/s41467-024-47762-y (PMC11035633; doi:10.1038/s41467-024-47762-y)
Supplement: Supplementary file 3 — Description of Additional Supplementary Files [file 41467_2024_47762_MOESM3_ESM.pdf]

Description of Additional Supplementary Files

File Name: Supplementary Data 1
Description: Mouse strains and additional statistics of Allen bulk neurotracing data for mapping cortical interhemispheric connections

File Name: Supplementary Data 2
Description: Experiments information of Allen bulk neurotracing data for different layers.

File Name: Supplementary Data 3
Description: The abbreviations and full names of brain regions.

File Name: Supplementary Data 4
Description: The neuron ID information for used neuronal illustration.

File Name: Supplementary Data 5
Description: Statistical test results for morphological analyses of neurons.

File Name: Supplementary Data 6
Description: Summary of widefield imaging sessions and duration of brain states (Awake, NREM, and REM) per session.

File Name: Supplementary Data 7
Description: T statistic results and p values involved in all t-tests.

File Name: Supplementary Data 8
Description: The median values, 25th percentile values, 75th percentile values, minimum values, maximum values, and p values for multiple comparisons are involved in all violin plot figures.
