# Supplementary material for: Diverse and asymmetric patterns of single-neuron projectome in regulating interhemispheric connectivity
Source: Nat Commun. 2024 Apr 22;15:3403. doi: 10.1038/s41467-024-47762-y (PMC11035633; doi:10.1038/s41467-024-47762-y)
Supplement: Supplementary file 5 — Reporting Summary [file 41467_2024_47762_MOESM5_ESM.pdf]

nature portfolio

Corresponding author(s): Cirong Liu

Last updated by author(s): Mar 18, 2024

Reporting Summary

Nature Portfolio wishes to improve the reproducibility of the work that we publish. This form provides structure for consistency and transparency in reporting. For further information on Nature Portfolio policies, see our Editorial Policies and the Editorial Policy Checklist.

Statistics

For all statistical analyses, confirm that the following items are present in the figure legend, table legend, main text, or Methods section.

n/a	Confirmed	
☐	☒	The exact sample size (n) for each experimental group/condition, given as a discrete number and unit of measurement
☐	☒	A statement on whether measurements were taken from distinct samples or whether the same sample was measured repeatedly
☐	☒	The statistical test(s) used AND whether they are one- or two-sided<br>*Only common tests should be described solely by name; describe more complex techniques in the Methods section.*
☐	☒	A description of all covariates tested
☐	☒	A description of any assumptions or corrections, such as tests of normality and adjustment for multiple comparisons
☐	☒	A full description of the statistical parameters including central tendency (e.g. means) or other basic estimates (e.g. regression coefficient) AND variation (e.g. standard deviation) or associated estimates of uncertainty (e.g. confidence intervals)
☐	☒	For null hypothesis testing, the test statistic (e.g. F, t, r) with confidence intervals, effect sizes, degrees of freedom and P value noted<br>*Give P values as exact values whenever suitable.*
☒	☐	For Bayesian analysis, information on the choice of priors and Markov chain Monte Carlo settings
☒	☐	For hierarchical and complex designs, identification of the appropriate level for tests and full reporting of outcomes
☐	☒	Estimates of effect sizes (e.g. Cohen's d, Pearson's r), indicating how they were calculated

Our web collection on statistics for biologists contains articles on many of the points above.

Software and code

Policy information about availability of computer code

Data collection	Allen bulk tracing data was freely accessible at http://connectivity.brain-map.org/.<br>ION single-neuron data was freely accessible at https://mouse.braindatacenter.cn/.<br>All mouse wide-field imaging data were collected using the lab's custom wide-field imaging system.<br>The imaging system used an Olympus MVX10 macro zoom microscope frame, equipped with an MVPLAPO 1X objective lens (Olympus).<br>The system was illuminated by a triple-wavelength LED light source (pE-300 ultra, CoolLED) including multiple TTL interfaces and a high-performance three-pass filter (69401, Chroma) for swift and stable switching between color lasers at high speeds.<br>The TTL signal from the camera was transmitted to an electrophysiological signal amplifier (Apollo, Jiangsu EGG Biotechnology Co., Ltd.).<br>The LED light source was synchronized with the camera using a high-frequency multi-channel trigger box (MultiStream Pro, CAIRN).<br>The acquisition system employed a high-resolution back-illuminated research-grade CMOS camera (Prime BSI Express, Teledyne).<br>Data were acquired by the Apollo Neural Recording System with a sample rate of 1000 Hz and low-pass filtering below 200 Hz.A Fast Fourier Transform was applied to the EEG/EMG data and the data were downsampled to one sample point per 5 seconds.
Data analysis	MATLAB R2019b, MATLAB R2020a and Python3.8 for wide-field imaging, single-neuron data and bulk tracing data preprocess, analysis and plotting; MATLAB R2020a for model simulation and plotting.<br>The codes for the analysis of wide-field imaging data (FC matrix, FC strength and stability), single-neuron data and bulk tracing data in this study are provided with the paper in the supplementary information.

For manuscripts utilizing custom algorithms or software that are central to the research but not yet described in published literature, software must be made available to editors and reviewers. We strongly encourage code deposition in a community repository (e.g. GitHub). See the Nature Portfolio guidelines for submitting code & software for further information.

Data

Policy information about availability of data

All manuscripts must include a data availability statement. This statement should provide the following information, where applicable:

- Accession codes, unique identifiers, or web links for publicly available datasets
- A description of any restrictions on data availability
- For clinical datasets or third party data, please ensure that the statement adheres to our policy

The Allen population tracing data are from public database via https://connectivity.brain-map.org.

The ION single-neuron tracing data are from the Brain Science Data Center of Chinese Academy of Sciences via https://mouse.digital-brain.cn/projectome/pfc.

The wide-field imaging data after basic processing(motion correction, hemodynamics regression and CCFv3 registration) are available via dataset DOI: 10.57760/sciencedb.17064.

Research involving human participants, their data, or biological material

Policy information about studies with human participants or human data. See also policy information about sex, gender (identity/presentation), and sexual orientation and race, ethnicity and racism.

Reporting on sex and gender	N/A
Reporting on race, ethnicity, or other socially relevant groupings	N/A
Population characteristics	N/A
Recruitment	N/A
Ethics oversight	N/A

Note that full information on the approval of the study protocol must also be provided in the manuscript.

Field-specific reporting

Please select the one below that is the best fit for your research. If you are not sure, read the appropriate sections before making your selection.

☒ Life sciences ☐ Behavioural & social sciences ☐ Ecological, evolutionary & environmental sciences

For a reference copy of the document with all sections, see nature.com/documents/nr-reporting-summary-flat.pdf

Life sciences study design

All studies must disclose on these points even when the disclosure is negative.

Sample size	No statistical method was used to predetermine sample size.<br><br>Allen bulk tracing data: 127 cortical injections from wildtype mice and 787 injections from 26 Cre-line transgenic mice were used to map the cortical neuronal projections across different regions and layers.<br><br>ION single-neuron projectome data: The data mapped axon projections of a total of 6,357 neurons in multiple brain regions, including frontal pole (FRP), orbitofrontal (ORB), prelimbic (PL), infralimbic (ILA), anterior cingulate (ACA), dorsal and ventral agranular insular (AId/v) and secondary motor (MOs) areas. Among the 6,357 neurons, dendrites of 1920 neurons were also reconstructed.<br><br>Wide-field imaging data: In total, 19 sessions of wide-field imaging recording from 8 male mice were collected. For each session, the mice underwent a different duration of awake, NREM, and REM sleep. Detailed information for each session is provided in Table S4.<br><br>Sample size was considered sufficient based on the high reproducibility of results and the comprehensive coverage of the brain regions of interest for our study.
Data exclusions	No data were excluded from the analysis.
Replication	Under each set of given model parameters, we applied numerous random simulations with different initial states made sure that the model results were reproducible: 500 times for modeling the relationship between heterogeneity and homotopic FC in Fig.3 and 1000 times for modeling the relationship between heterogeneity and asynchronous periods duration, FC strength and FC variability in Fig.4.

Randomization	All animals were normal subjects and belonged to one health group, so randomization of animals to different groups is not relevant to our study design.
Blinding	As the animals were not divided into different groups, blinding were not applied to this study.

Reporting for specific materials, systems and methods

We require information from authors about some types of materials, experimental systems and methods used in many studies. Here, indicate whether each material, system or method listed is relevant to your study. If you are not sure if a list item applies to your research, read the appropriate section before selecting a response.

Materials & experimental systems

n/a	Involved in the study
☒	☐ Antibodies
☒	☐ Eukaryotic cell lines
☒	☐ Palaeontology and archaeology
☐	☒ Animals and other organisms
☒	☐ Clinical data
☒	☐ Dual use research of concern
☒	☐ Plants

Methods

n/a	Involved in the study
☒	☐ ChIP-seq
☒	☐ Flow cytometry
☒	☐ MRI-based neuroimaging

Animals and other research organisms

Policy information about studies involving animals; ARRIVE guidelines recommended for reporting animal research, and Sex and Gender in Research

Laboratory animals	For wide-field imaging, 8 Thy1-GCaMP6s transgenic adult male mice (JAX024275) were procured from the Jackson Laboratory and then bred in the SPF class mouse house at the Center of Excellence in Brain Science and Intelligent Technology, Chinese Academy of Sciences. Mice were housed under strict conditions at 20~26 °C, with daily temperature difference being less than or equal to 4 °C, and 30~70 % relative humidity (normally around 60%). We started wide-field imaging experiments on each mouse since the age of at least 12 weeks, and ended at the age of about 5~6 months.
Wild animals	This study did not involve wild animals.
Reporting on sex	The mouses for wide-field imaging are all male.
Field-collected samples	This study did not involve field-collected samples.
Ethics oversight	The experimental procedures were approved by the Laboratory Animal Care and Use Committees from the Center for Excellence in Brain Science and Intelligence Technology (Institute of Neuroscience) of the Chinese Academy of Sciences.

Note that full information on the approval of the study protocol must also be provided in the manuscript.
